# Effect of liquid surface area on hydrogen sulfide oxidation during micro-aeration in dairy manure digesters

**DOI:** 10.1371/journal.pone.0185738

**Published:** 2017-10-04

**Authors:** Walter Mulbry, Kaitlyn Selmer, Stephanie Lansing

**Affiliations:** 1 Agricultural Research Service, United States Department of Agriculture, Beltsville, Maryland, United States of America; 2 Department of Environmental Science and Technology, University of Maryland, College Park, Maryland, United States of America; The University of Akron, UNITED STATES

## Abstract

Although there are a variety of commercially available biological and chemical treatments for removal of hydrogen sulfide (H_2_S) from biogas, managing biogas H_2_S remains a significant challenge for agricultural digesters where labor and operational funds are very limited compared to municipal and industrial digesters. The objectives of this study were to evaluate headspace aeration for reducing H_2_S levels in low cost plug flow digesters and to characterize the relationship between the liquid surface area and H_2_S oxidation rates. Experiments with replicate field scale plug flow digesters showed that H_2_S levels decreased from 3500 ppmv to <100 ppmv when headspace oxygen levels were 0.5 to 1%. Methane production was not affected by aeration rates that resulted in headspace oxygen levels of up to 1%. Pilot scale experiments using 65 to 104 L desulfurization units showed that H_2_S oxidation rates increased with increases in liquid surface area. These results support the hypothesis that H_2_S oxidation rates are limited, in part, by the surface area available for oxygen transfer, and can be increased by growth of biofilms containing H_2_S oxidizing bacteria. Maximum removal rates corresponded to 40 to 100 g S m^-2^ d^-1^ of liquid surface area at biogas retention times of 30 to 40 min.

## Introduction

Untreated manure from animal operations can result in noxious odors and the unintentional release of the greenhouse gas methane (CH_4_). However, when properly treated in an anaerobic digester, animal waste can be transformed into environmental and economic benefits: 1) the captured methane becomes a source of renewable energy, 2) emissions of methane and noxious odors are sharply reduced, and 3) the fertilizer value of the manure increases as a portion of organic N is converted to plant available ammonia-N increases [1].

Biogas from manure digesters usually contains 55 to 65% CH_4_, 35 to 45% CO_2_, and 0.1 to 0.4% of hydrogen sulfide (H_2_S) [2]. In addition to its toxicity and reactivity with metals and cement, H_2_S is readily converted into SO_2_ and H_2_SO_4_, which are also highly corrosive. Although there are a variety of commercially available biological and chemical treatments for removal of H_2_S from biogas (recently reviewed by [2,3], all require some level of chemical or water inputs and maintenance. In practice, managing biogas H_2_S remains a significant challenge for agricultural digesters where labor and operational funds are very limited compared to municipal and industrial digesters. As an alternative to treat biogas, relatively low volumes of air (or oxygen) can be injected into the digester headspace. The basis of this method lies in the presence of sulfide-oxidizing bacteria in the manure feedstock [4] that convert dissolved sulfide into S^o^ and SO_4_^2-^ [5]. The feasibility of H_2_S oxidation under micro-aerobic conditions has been demonstrated in pilot- scale experiments (recently reviewed by [2,3]) and by monitoring of large scale sewage sludge digesters [6]. However, further research is needed to transfer these findings to practice on plug-flow agricultural digesters where biogas composition and aeration systems are monitored infrequently, if at all.

There were two specific objectives of this study. The first objective was to evaluate headspace aeration for reducing hydrogen sulfide (H_2_S) levels in low cost plug flow manure digesters. Six field-scale Taiwanese-model digesters were used to determine the effect of aeration rate on H_2_S concentrations and methane production under field conditions. The second objective was to determine the relationship between the liquid surface area in digesters and H_2_S oxidation rates during headspace aeration. We hypothesized that H_2_S oxidation rates were limited, in part, by the surface area available for growth of biofilms containing H_2_S oxidizing bacteria. In this part of the study, we used replicate pilot-scale desulfurization units [7] that were operated at a range of H_2_S loading rates.

## Materials and methods

### Substrate

Dairy manure was obtained from the USDA’s Dairy Research Unit within the Beltsville Agricultural Research Center (BARC) (Beltsville, Maryland, USA). The dairy’s free stall barn houses approximately 100 dairy cows and uses sawdust as bedding on top of rubber pillows. Manure is mechanically scraped into holding pits and pumped daily from the pits to a FAN screw-press solid separator system. The solids are collected and composted, and the solids-separated liquid manure (containing roughly 3% TS) is pumped over the course of a day into the full scale BARC digester (540 m^3^ total volume, 400 m^3^ working volume) for treatment. During the course of this study, the BARC digester operated without mixing at temperatures ranging from 30 to 35°C with biogas pressure of 2000 to 2500 Pa (20–26 cm of water) and typically produced 65 m^3^ d^-1^ of biogas containing 63 to 66% CH_4_, 32 to 34% CO_2_, 2000 to 4000 parts per million by volume (ppmv) H_2_S.

### Field-scale anaerobic digesters

**Fig 1:**
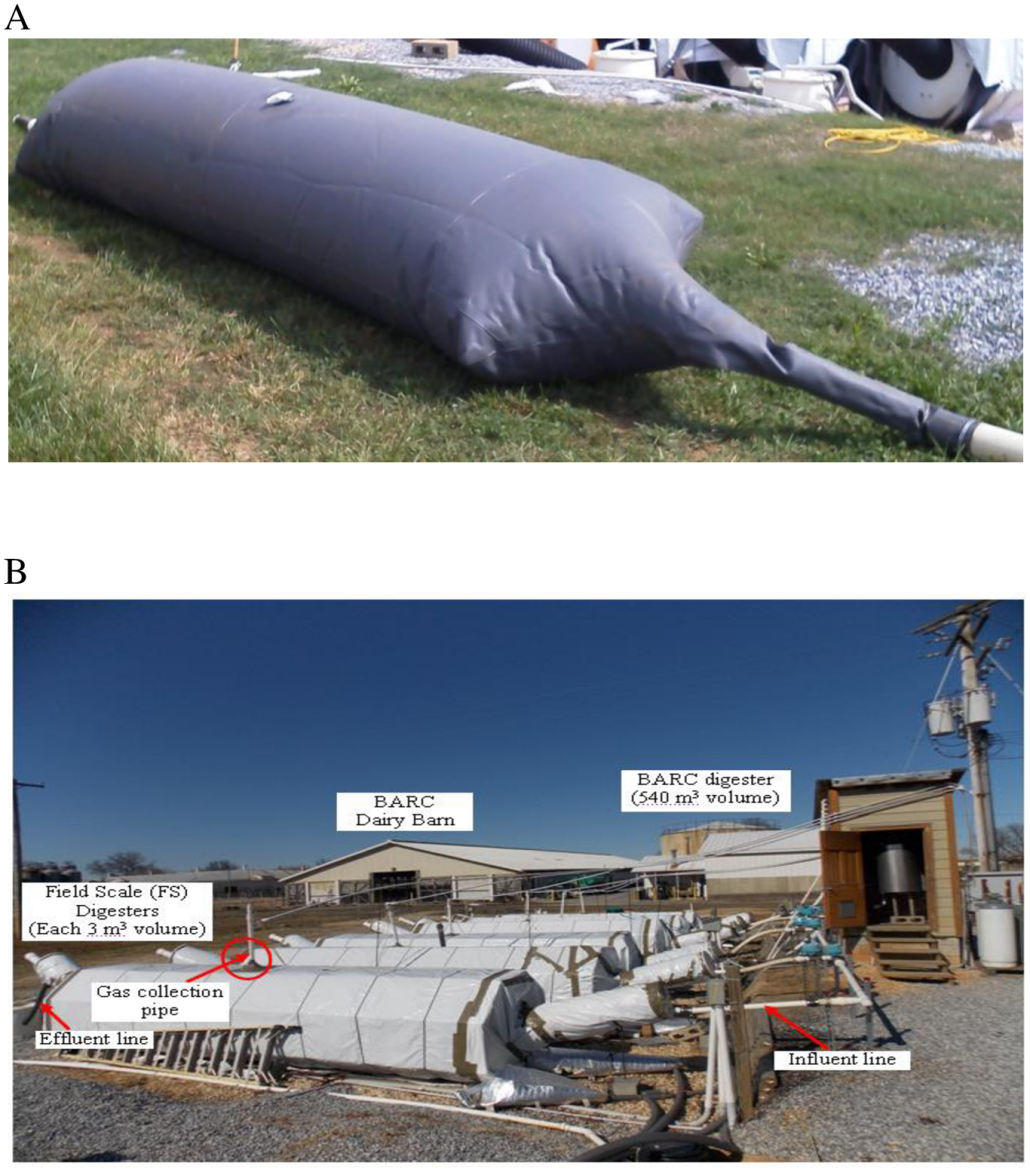
Images of the six plug-flow field-scale (FS) digesters used in this study. A, Digester bag prior to installation. The biogas vent tube (10 cm diameter) is not shown but is located in the top center of the bag. B, Field site overview, with the digester bags placed inside insulated HDPE corrugated drainage pipes.

Anaerobic digestion experiments were carried out using six modified Taiwanese-model field-scale (FS) digesters (Fig 1) at the BARC dairy [8,9]. Each FS digester is composed of a tapered 5.2 m long, 0.9 m diameter PVC-based digester bag with a 1.0 mm thick membrane. The digester bags were placed inside 1.1 m diameter corrugated high-density-polyethylene (HDPE) drainage pipes for protection and insulation. Each FS digester has a total capacity of 3 m^3^ and was operated at a liquid capacity of 67% (2 m^3^ working volume), with 33% headspace for biogas collection. The FS digesters are plug-flow reactors and were operated without mixing. Digesters were maintained at 28 ± 2°C by circulating a heated 30% glycol solution through heat exchanger material located between the digester bags and foam insulation. Pumps, valves and heat ignition were controlled electronically through a Labview^™^ software program (National Instruments Corp., Austin, TX, USA).

Prior to the study, each FS digester was filled with 1 m^3^ of inoculum from the BARC farm digester and 1 m^3^ of separated manure. Each digester was subsequently loaded with 160 L d^−1^ separated manure, corresponding to an organic loading rate of 1.5 kg VS m^−3^ d^−1^) and hydraulic retention time (HRT) of 17 days. Correspondingly, each digester produced 160 L d^−1^ of digested effluent that was collected in a central sump prior to transfer back to the farm’s manure lagoon. The amount of the manure solids was chosen to keep the digester organic loading rate (OLR) within the suggested range of 1 to 3 kg VS m^−3^ d^−1^ [10]. Steady state conditions, defined as stable methane production and methane content, was reached in the digesters after they were operated for two hydraulic retention times (HRT) (data not shown).

After achieving steady state conditions, randomly chosen pairs of digesters were assigned for one of three treatments (no aeration, low rate aeration (1 to 5% of air to biogas (v/v), or high rate aeration (5 to 10% of air to biogas (v/v), and operated for 18 weeks (7.4 HRTs). For digesters chosen for headspace aeration, air was pulsed 20 times per day into the headspace of each digester using an electronic timer (model 50015, Woods/Coleman Cable, Waukegan, IL) and an aquarium pump (model 9902, Petco, San Diego, CA) that delivered air to two outlets that floated on the surface of the digestate.

### Pilot-scale desulfurization experiments

Polypropylene barrels (Uline, Pleasant Prairie, WI) containing digestate from the farm scale BARC digester were used as pilot-scale desulfurization units to remove H_2_S from BARC digester biogas. In the first set of experiments, sealed 65 L barrels (35 cm diameter, 60 cm height) containing 6 L of digestate (59 L headspace) were used to determine the relationship between liquid surface area and H_2_S oxidation rates. Pairs of barrels were operated vertically or horizontally with liquid surface areas of 0.099 and 0.150 m^2^, respectively. In the second set of experiments, pairs of open top 104 L barrels (43 cm diameter, 71 cm height, 104 L) with removable gas tight lids and containing 30 L of digestate (74 L headspace) were used in experiments in which we attempted to increase H_2_S oxidation capacity by increasing the wetted surface area for biofilm growth. In one pair of barrels, a vertical piece of cotton terrycloth fabric (29 X 29 cm) was suspended vertically into and above the digestate surface in each barrel. The fabric was saturated with digestate at the beginning of the experiment. Another pair of barrels was operated without the addition of fabric. In both experiments, biogas from the BARC digester was continuously introduced into the headspace of each barrel (approximately 5 cm above the digestate surface) at rates ranging from 0.1 to 3 L min^-1^. Ambient air was continuously pumped into the headspace of the barrels using small aquarium pumps (described above) at rates corresponding to 5% (v/v) of the biogas flow rate (ranging from 5 to 150 ml min^-1^).

Barrels were insulated using 2" closed foam insulation board (R-10) underneath the barrels and 2" fiberglass insulation (R-13) wrapped around the sides. Barrels were maintained at temperatures between 20 to 25°C using 50 W heating pads taped to the sides of the barrels (model 756–500, Sunbeam, Boca Raton, FL). Flow rates of biogas and air were determined daily using a mass flow meter (model GFM 17, Aalborg Instruments) and rotameter (Dwyer Instruments), respectively.

### Analytical methods

Biogas produced by the FS digesters was monitored using low-pressure cumulative gas meters (model PGM.75, EKM Metering, Santa Cruz, CA, USA). Although biogas readings were recorded daily, biogas production values were calculated using weekly averages. Gas composition (CH_4_, CO_2_, O_2_, H_2_S) of biogas from FS digesters, biogas the BARC digester, and treated biogas from each desulfurization unit were determined daily using a portable gas analyzer (Biogas 5000, Landtec Instruments, Dexter, MI). The instrument was calibrated using gas standards provided by the manufacturer.

Influent and effluent samples from the FS digesters were collected weekly and analyzed for total solids (TS), volatile solids (VS), pH, and alkalinity according to Standard Methods [11]. Digestate samples from desulfurization units were collected only at the beginning and end of the experiments.

### Statistical methods

Analysis of variance (ANOVA) was performed to test the significance among treatments for maximum H_2_S oxidation rate (g S d^-1^ m^-2^). Post-hoc pairwise comparisons of treatments were conducted using Tukey’s Honest Significant Difference test (R 3.1.2; 95% confidence level) [12].

## Results and discussion

### Use of field-scale digesters to determine the effect of aeration rate on biogas H_2_S concentrations and CH_4_ production

Six field-scale digesters were used to determine the effect of aeration rate on hydrogen sulfide concentrations and methane production under field conditions (Fig 1). After steady state conditions were achieved, the FS digesters were operated at 28°C using solids separated dairy manure. The HRT and OLR were 17 days and 1.5 ± 0.1 kg VS m^3^ d^-1^, respectively, during the study for all digesters. The two non-aerated digesters produced 0.9 to 1.7 m^3^ biogas per day, corresponding to biogas retention times of 14 to 26 hours. The mean methane production rate from the non-aerated digesters was 0.45 ± 0.02 m^3^ d^-1^ m^-3^ and specific methane production value was 0.32 ± 0.01 m^3^ CH_4_ d^-1^ kg^-1^ VS. Biogas produced from the non-aerated digesters contained CH_4_ concentrations of 62% to 68%, CO_2_ concentrations of 32% to 37%, and H_2_S concentrations of 2900 to 4500 ppmv over the 18 week study period (not shown).

**Fig 2:**
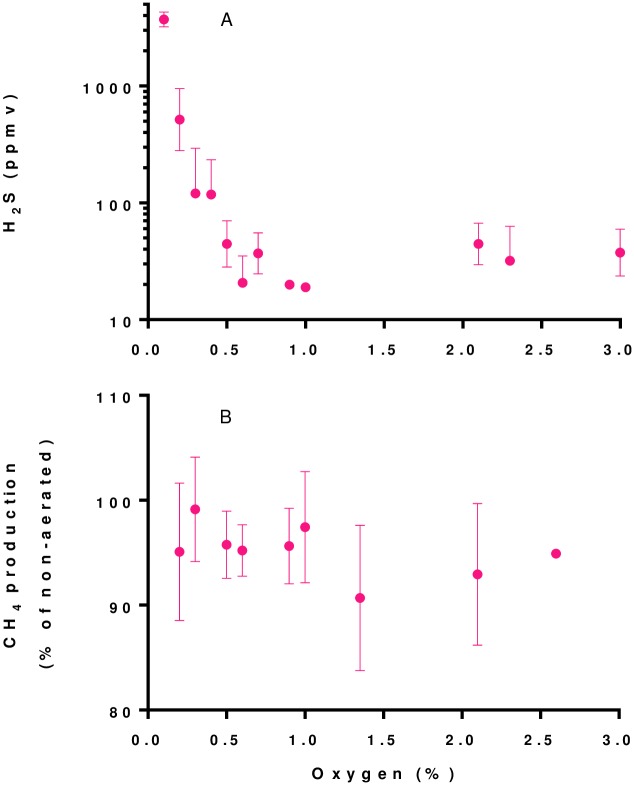
Hydrogen sulfide concentration and methane production as a function of residual oxygen concentrations in micro-aerated FS digesters. A, Influence of aeration on H_2_S concentrations in biogas. Values are means of 2 to 12 daily measurements from duplicate aerated field scale digesters operated for 18 weeks. During this period, H_2_S concentrations from duplicate non-aerated digesters ranged from 3000 to 4000 ppmv with a mean value of 3570 ppmv. Vertical bars show standard error values (three error bars are clipped at the axis limit). B, Effect of aeration on CH_4_ production. Values are calculated from weekly means from duplicate aerated field digesters operated with and without aeration. Vertical bars show standard error values.

Results showed that increased levels of aeration of digester headspace resulted in decreased levels of H_2_S. Two pairs of digesters were chosen to receive aeration at different rates over an 18-week period. Another pair of digesters did not receive aeration. Air was pulsed into the headspace (20 pulses per day) as it was difficult to accurately maintain the low air flow rates needed for continuous aeration. At aeration rates corresponding to 2% to 10% of air per volume of biogas, residual oxygen levels in the biogas ranged from 0.1 to 3%. Although we predicted that any amount of excess oxygen would indicate complete oxidation of H_2_S, H_2_S levels in the biogas were consistently less than 500 ppmv only when residual oxygen levels within the biogas were ≥ 0.3% (v/v) (Fig 2, panel A). H_2_S levels in the biogas were consistently less than 100 ppmv only when residual oxygen levels within the biogas were ≥ 0.5% (v/v). These results are consistent with those from other studies using sewage sludge digesters. Hydrogen sulfide removal efficiencies of <99% have been reported from digesters with biogas retention times > 5 hr (recently reviewed by [2,3]).

Aeration of digester headspace did not significantly affect CH_4_ production. As expected, CH_4_ and CO_2_ concentrations in the biogas decreased with increasing levels of aeration because of dilution with air (primarily nitrogen). However, mean methane production values from aerated digesters (0.43 ± 0.01 m^3^ CH_4_ d^-1^) were not significantly different (p = 0.277) than values from digesters operated without aeration (0.45 ± 0.02 m^3^ CH_4_ d^-1^). There was no apparent trend with regard to the effect of aeration on CH_4_ production up to levels yielding biogas oxygen concentrations up to 1% (Fig 2, panel B). With respect to the effect of biogas oxygen concentrations above 1%, the results are less clear because of relatively few (and variable) results under these conditions. There were no differences in the pH or alkalinity values of effluents from digesters receiving aeration compared those not receiving aeration (results not shown). We did not measure sulfur concentrations in digester effluents because we expected that much of the elemental sulfur precipitate would be retained with solids in these unmixed digesters [9]. There are few results from other studies with which to compare our results because most studies have used single digesters operated under different conditions through time rather than replicate digesters operated simultaneously. However, effects of micro-aeration on CH_4_ production have been considered to be minimal or slightly positive [3,6].

### Use of pilot-scale desulfurization units to determine the effect of wetted surface area on H_2_S oxidation rates during micro-aeration

Although H_2_S levels in the FS digesters (with relatively long biogas retention times of 14 to 26 hours) were significantly reduced by aeration, we sought to determine the effectiveness of aeration under a range of biogas flow rates and much lower retention times. In addition, since the majority of H_2_S oxidation within digesters is likely due to microbial biofilms located on the digestate surface, we also sought to determine the effect of varying the available liquid or wetted surface area on sulfur oxidation rates during headspace aeration. For these experiments, we made minor modifications to a 10 L desulfurization unit described by Ramos and coworkers [7]. In the first set of experiments, we determined H_2_S oxidation rates as a function of H_2_S loading rate using polypropylene barrels with two different liquid surface areas. Pairs of 65 L barrels containing digestate were operated vertically (0.099 m^2^ liquid surface area) or horizontally (0.150 m^2^ liquid surface area) and subjected to micro-aeration under different flow rates of biogas from the BARC digester.

**Fig 3:**
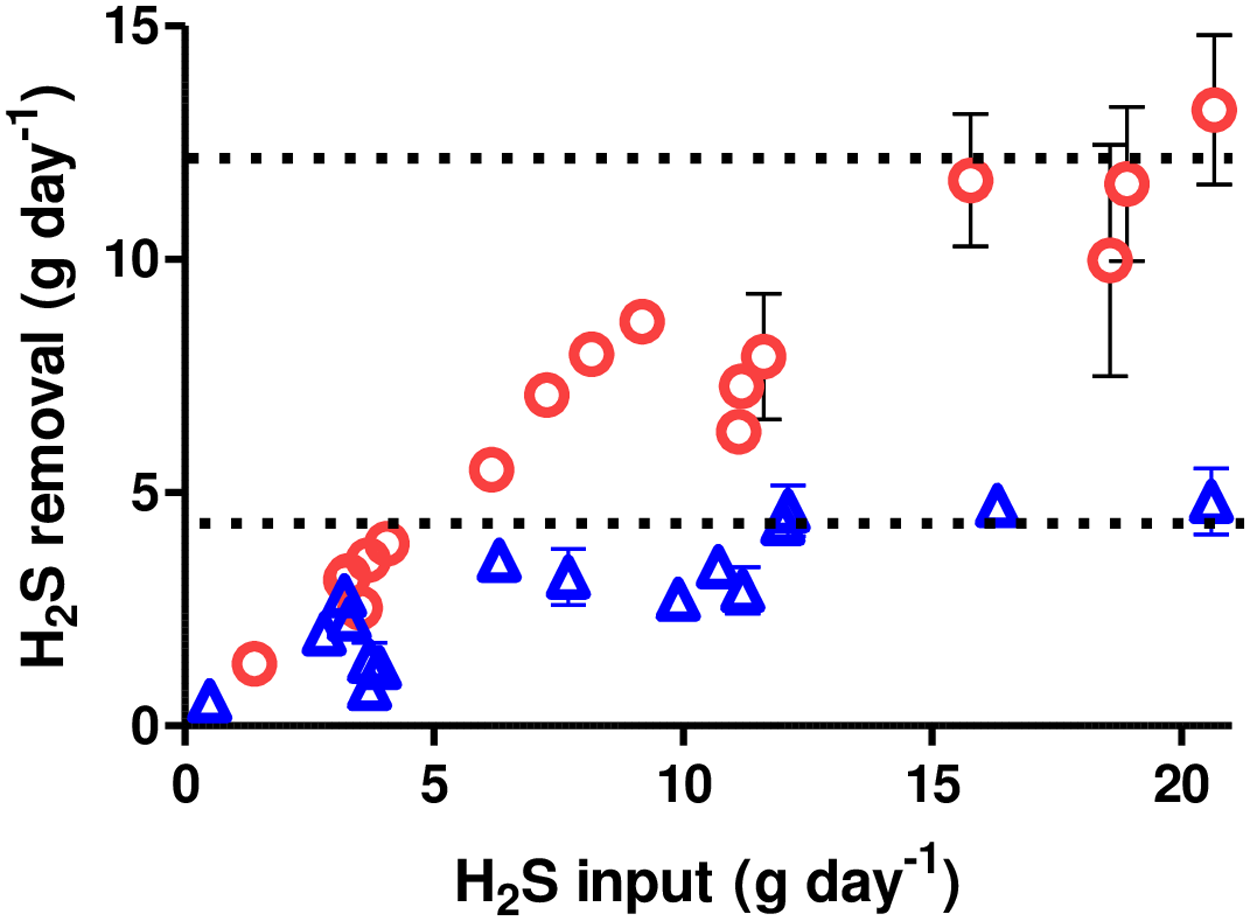
Hydrogen sulfide oxidation rates as a function of H_2_S loading rate in vertical and horizontal 65 L barrels. Values are means of daily measurements from duplicate aerated barrels. Red circles, horizontal barrels. Blue triangles, vertical barrels. Vertical bars show standard error values. Dotted lines show estimated maximum removal rates corresponding to values shown in Table 1.

Results show that the horizontal tanks (containing 50% more liquid surface area compared to the vertical tanks) oxidized H_2_S at maximum rates that were approximately 3-fold higher than the vertical barrels (Fig 3, Table 1). When normalized for surface area, maximum H_2_S oxidation rates from horizontal units are approximately 2-fold higher than those from the vertical units (Table 1). Since the barrels were otherwise identical, we believe that the likely explanation for the different rates is that increased liquid/gas surface area leads to more surface biofilm containing H_2_S oxidizing bacteria and higher rates of H_2_S oxidation. There are few results from other studies with which to compare our results because most studies have not been designed to determine maximum H_2_S oxidation rates and few provide information needed to determine digester liquid surface area. The desulfurization unit described by Ramos and coworkers reduced the effluent biogas H_2_S content from approximately 3000 ppmv to between 100 and 200 ppmv [7]. Under their conditions, the calculated H_2_S oxidation rate was 25 g S d^-1^ m^-2^. This value is within the range of values determined in our study (Table 1). Nghiem and coworkers conducted a study in which micro-aeration was controlled by oxidation reduction values in the digestate [13]. Under their conditions, effluent biogas H_2_S concentrations decreased from approximately 6000 ppmv to 30 ppmv and the calculated H_2_S oxidation rate was 2.6 g S d^-1^ m^-2^ (Table 1). This value is lower than values determined in this study but is likely an underestimate of the maximum rate because their study was not designed for this purpose.

**Table 1:** Comparison of H_2_S oxidation rates using pilot-scale desulfurization units.

	65 L vertical barrel	65 L horizontal barrel	104 L barrel no fabric	104 L barrel with fabric	10 L desulfur-ization unit [7]^1^	50 L digester [13]^2^
Headspace volume (L)	59	59	74	74	9	20
Liquid/wetted surface area (m^2^)	0.099	0.150	0.146	0.224	0.049	0.126
Biogas H_2_S (ppmv)	3500	3500	3500	3500	3000	6000
Biogas H_2_S (g m^-3^)	4.88	4.88	4.88	4.88	4.18	8.38
Biogas flow rate at maximum H_2_S oxidation rate^2^ (LPM)	1.5	1.5	2.0	2.5	0.15	0.027
Biogas retention time (min)	39	39	37	30	60	740
Maximum H_2_S oxidation rate^3^(g S d^-1^)	4.3 ± 0.3	12.4 ± 1.3	15.3 ± 0.5	22.4 ± 0.3	n.d. ^4^	n.d. ^4^
Maximum H_2_S oxidation rate^3^(g S d^-1^ m^-2^)	43 ± 3a	83 ± 7b	104 ± 5c	101 ± 2bc	n.d. ^4^	n.d. ^4^
Maximum H_2_S input rate for effluent content <500 ppmv H_2_S (g S d^-1^ m^-2^)	n.d.	24 (29%)^5^	27 (26%)	44 (43%)	25	n.d. ^4^
Maximum H_2_S input rate for effluent content <100 ppmv H_2_S (g S d^-1^ m^-2^)	n.d.	6.6 (8%)^4^	9.3 (9%)	18 (18%)	n.d.	2.6

^1^Experiments used a 10 L (25 cm diameter) plastic container and achieved effluent biogas H_2_S values of 100 to 200 ppmv. Maximum H_2_S oxidation rates were not determined.

^2^Experiments used a 50 L (40 cm diameter) digester and achieved effluent biogas H_2_S values of 30 ppmv. Maximum H_2_S oxidation rates were not determined.

^3^Values are means ± SE of 6 or 7 daily measurements from duplicate barrels. Treatment means with different letters are statistically significant at the 0.05 significance level.

^4^n.d.: not determined.

^5^Values in parentheses indicate theoretical H_2_S input rate with effluent content <500 ppmv or < 100 ppmv H_2_S as a percentage of the maximum oxidation rate.

**Fig 4:**
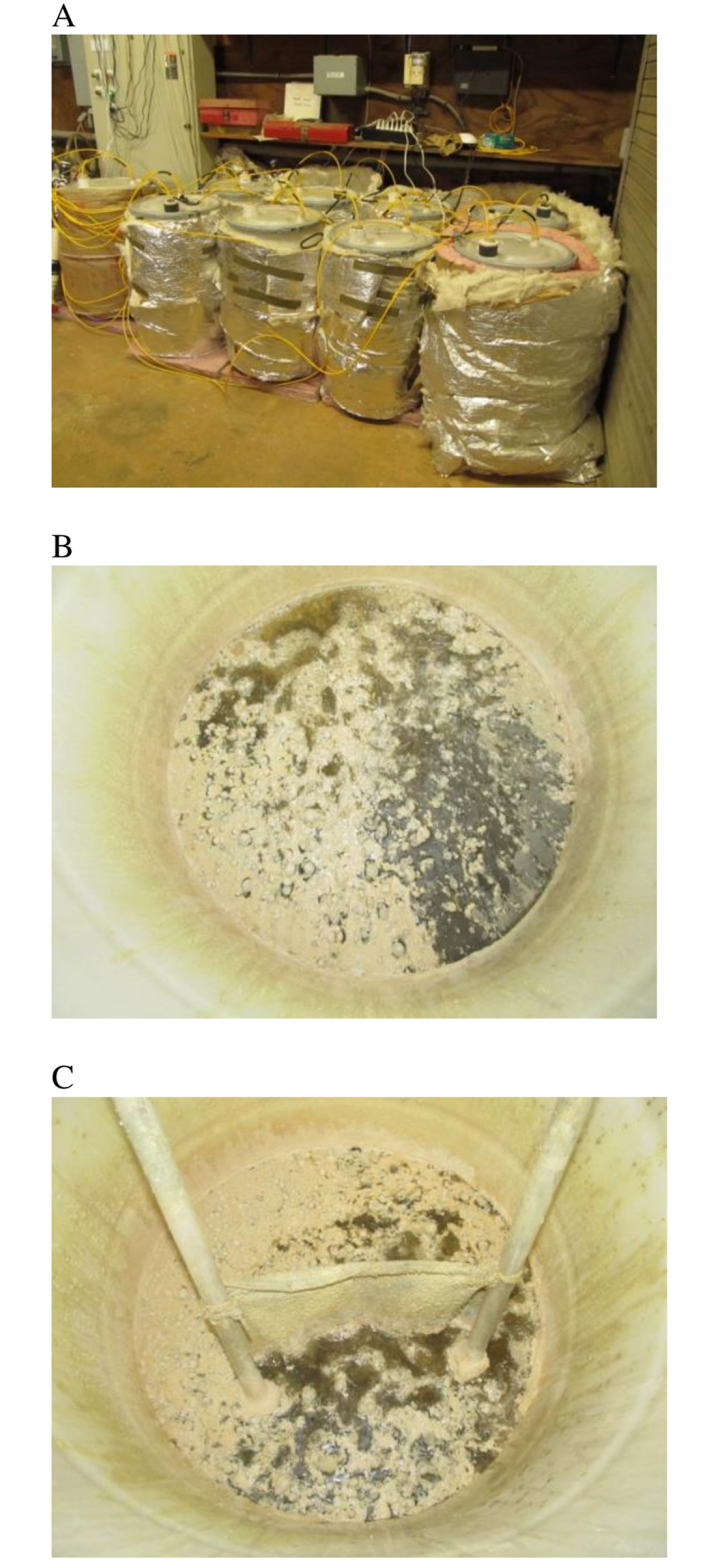
Images of 104 L micro-aeration units. A, View of insulated barrels. B, View of sulfur deposition on digestate surface in barrel receiving aeration. C, View of saturated fabric (29 x 29 cm) in barrels receiving aeration.

In the second set of experiments, we determined the effect of increasing the wetted area for biofilm growth on the H_2_S oxidation rate. In these experiments, we determined H_2_S oxidation rates using 104 L barrels containing digestate (Fig 4, panel A). A vertical piece of cotton terrycloth fabric (29 X 29 cm) was suspended vertically into and above the digestate surface in each of a pair of barrels (Fig 4, panel C). The fabric was saturated with digestate at the beginning of the experiment. A second pair of barrels was operated without fabric (Fig 4, panel B). Barrels were subjected to different flow rates of biogas and aerated at 5% v/v of biogas flow as described above for 30 days.

**Fig 5:**
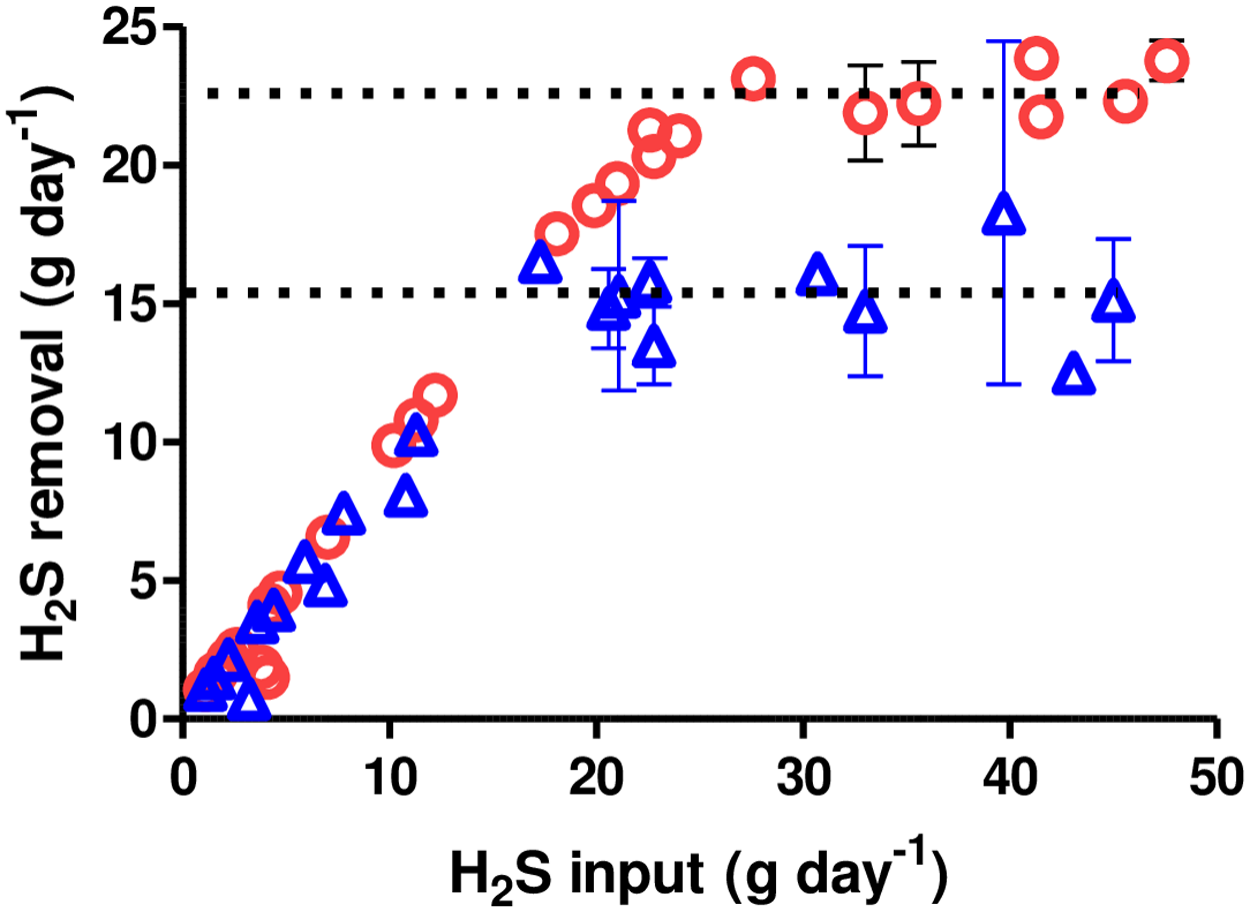
Hydrogen sulfide oxidation rates as a function of loading rate in 104 L barrels with and without vertical strips of cotton fabric. Blue triangles, barrels without fabric. Red circles, barrels with saturated cotton fabric in headspace above digestate. Values are means of daily measurements from duplicate aerated barrels. Vertical bars show standard error values. Dotted lines show estimated maximum removal rates corresponding to values shown in Table 1.

Results show that the units containing fabric (with approximately 50% more wetted surface area compared to the units without fabric) oxidized H_2_S at maximum rates that were approximately 50% higher than units without fabric (Fig 5, Table 1). When normalized for wetted surface area, maximum H_2_S oxidation rates from units with and without fabric were nearly identical (Table 1). Use of taller pieces of saturated fabric (29 cm wide x 59 cm high) in subsequent experiments did not result in increased rates of H_2_S oxidation (results not shown). It is possible that addition of wetted fabric above 29 cm did not lead to increased oxidation rates because fabric above this height did not maintain adequate moisture levels. Indeed, we observed that the fabric remained visibly wet only about 12 cm above the digestate surface.

Although the maximum oxidation rates measured in these experiments are useful in determining limiting factors in the micro-aeration process, digester operators are focused on the maximum H_2_S loading rates that achieve specific H_2_S target concentrations. Our results suggest that biogas H_2_S concentrations were reduced from approximately 3500 ppmv to less than 500 ppmv at H_2_S loading rates corresponding to approximately 25 to 40% of the maximum oxidation rates (Table 1). Biogas H_2_S concentrations were reduced to below 100 ppmv at H_2_S loading rates corresponding to 8 to 18% of the maximum oxidation rates.

Although digestates from desulfurization units turned yellow and contained visible flocs of sulfur-containing solids, we did not characterize sulfur recovery in these units. Alkalinity and pH values were lower in digestates from units receiving aeration compared to those not receiving aeration. However, we did not collect samples needed for determining a relationship between pH, alkalinity, and oxidized sulfur (samples were collected only at the beginning and end of experiments). Ramos and coworkers collected sulfur-containing solids in their study of a desulfurization unit and estimated that the recovered solids contained 60% of input H_2_S-S (assuming that all H_2_S-S was converted to S^0^) [7].

Our results suggest that maximum H_2_S oxidation rates during headspace aeration are limited, in part, by the wetted area available for biofilm growth and/or activity. Vertical saturated fabric above the digestate surface provides a suitable habitat for the biofilm activity. Although it may be possible to maximize oxidation efficiency further by reducing fabric height below 29 cm, results from experiments using shorter lengths of fabric were inconclusive (not shown). It is likely that H_2_S oxidation rates could be increased by addition of multiple strips of a durable absorbent material positioned vertically in the digester headspace (or in an external desulfurization unit).

Although the efficiency of micro-aeration is influenced by other factors (such as H_2_S concentration, biogas retention time, digestate temperature, and adequate mixing of oxygen at the digestate surface), the sulfur oxidation values based on liquid surface area may be useful for estimating H_2_S oxidization capacities of digesters and desulfurization units. Using 10 g S m^-2^ d^-1^ for the H_2_S oxidation rate needed to achieve biogas effluent containing <100 ppmv H_2_S, the field-scale horizontal plug-flow digesters (with a surface area of approximately 3.3 m^2^) could be operated to produce up to 33 g H_2_S d^-1^. This rate corresponds to a daily biogas production value of about 6 m^3^ d^-1^ (assuming a content of 3500 ppmv H_2_S (5 g S m^-3^)) that is 3-fold higher than our typical production rates of 1–2 m^3^ d^-1^ [9]. The sulfur oxidation values can also be used to estimate the size of an external desulfurization unit needed to treat digester biogas. For the farm-scale BARC digester, the estimated H_2_S-S output is approximately 325 g S d^-1^ (assuming 65 m^3^ d^-1^ biogas containing 3500 ppmv H_2_S). Using values of 25 or 10 g S m^-2^ d^-1^, external desulfurization units of approximately 13 m^2^ or 33 m^2^ would be required to achieve biogas H_2_S concentrations below 500 ppmv or below 100 ppmv, respectively.

A recent report documented the effectiveness of micro-aeration for biogas desulfurization in full scale digesters at seven wastewater treatment facilities [6]. Desulfurization efficiencies ranged from 74 to >99% using biogases that, when untreated, contained from 800 to 7500 mg m^-3^ H_2_S. Our results suggest that micro-aeration should also be effective in much simpler agricultural digester systems. Widespread use of this simple technology may be facilitated by the introduction of low cost oxygen probes coupled to air pumps that can be used to safely maintain low but adequate levels of oxygen (< 0.5%) within digester headspaces.

## Supporting information

S1 Appendix Datasets used for Figs 2, 3 and 5. (XLSX)
